# Ewww–Investigating the neural basis of disgust in response to naturalistic and pictorial nauseating stimuli

**DOI:** 10.3389/fpsyt.2022.1054224

**Published:** 2023-01-23

**Authors:** Gesa Berretz, Canan Dutschke, Elodie Leonard, Julian Packheiser

**Affiliations:** ^1^Department of Biopsychology, Institute of Cognitive Neuroscience, Faculty of Psychology, Ruhr University Bochum, Bochum, Germany; ^2^Social Brain Lab, Netherlands Institute for Neuroscience, Amsterdam, Netherlands

**Keywords:** lateralization, asymmetry, right hemisphere hypothesis, disgust, EEG

## Abstract

Emotion induction in psychological and neuroscientific research has been mostly done by presenting participants with picture or film material. However, it is debatable whether this passive approach to emotion induction results in an affective state comparable to real-life emotions, and if the neural correlates of emotion processing are ecologically valid. To investigate the appropriateness of pictures for the induction of emotions, we presented 56 participants in a within-subjects design with naturalistic disgusting and neutral stimuli as well as with pictures of said stimulus material while recording continuous EEG data. We calculated asymmetry indices (AIs) for alpha power as an index of emotion processing and emotion regulation at the F3/4, F5/6, F7/8, and O1/2 electrode pairs. Participants reported higher disgust ratings for disgusting naturalistic compared to disgusting pictorial stimuli. Investigating changes in the EEG signal in participants with a pronounced disgust response (*n* = 38), we found smaller AIs for naturalistic stimuli compared to pictures. Moreover, in this disgusted sub-sample, there were smaller AIs in response to naturalistic disgusting stimuli compared to pictorial disgusting and neutral stimuli at the O1/2 electrode pair indicating stronger activation of the right relative to the left hemisphere by naturalistic stimuli. As the right hemisphere has been shown to display dominance in processing negative and withdrawal-associated emotions, this might indicate that naturalistic stimuli are more appropriate for the induction of emotions than picture stimuli. To improve the validity of results from emotion induction, future research should incorporate stimulus material that is as naturalistic as possible.

## Introduction

Imagine coming home, hungry after a long trip and thus opening the fridge to get a snack. Opening the door, a foul stank hits you and you immediately see that it is coming from the moldy rotten cucumber you forgot to throw out. If this would happen in real life, most people would experience an intense feeling of disgust: an emotion characterized by revulsion and aversion (1).

Disgust is one of the six basic emotions (2) and is present across all cultures (3). More recent research has shifted to a more constructionist approach taking broadly distributed functional networks representing basic psychological operations as a basis for the production of a variety of emotional states (4, 5). The evolutionary benefit of disgust has been proposed to lie in the defense against parasitic diseases and the avoidance of stimuli associated with contamination (6, 7). Disgust can be differentiated in several dimensions, i.e., core disgust of feces, vomit, and vermin, blood-injury disgust, as well as interpersonal and sexual disgust of diseased individuals, and inappropriate sexual contact (8).

Using functional magnetic resonance imaging (fMRI), physical disgust has been identified to be strongly associated with the activation of the insula (9, 10). However, activation in the insular cortex is not specific to the experience of disgust (11), lending evidence to the view that the emergence of different emotions relies on similar brain networks (12). Moreover, the bilateral amygdala, the right inferior occipital and inferior frontal gyrus, left inferior temporal gyrus, as well as the left superior frontal gyrus have been shown to respond to disgusting stimuli (13). Disgust related to pathogens specifically was related to activation of the left amygdala, fusiform gyrus, and inferior frontal gyrus as well as the bilateral orbitofrontal cortex and precuneus (14). A number of studies have also investigated the neural basis of disgust using electroencephalography (EEG). Investigating resting state EEG, Li et al. (15) found that higher disgust sensitivity was associated with EEG microstate activity indicative of activation of the anterior default mode network that is involved in executive control. Task-related designs found increased amplitudes in event-related potentials (ERPs) such as the early posterior negativity (EPN), the anterior P2 component or the late positive potential (LPP) when comparing disgusting to fearful or neutral stimuli (16, 17). A recent study by Revers et al. (18) used differential responses in classically affect-associated components such as the N1, P2, EPN, or LPP, to classify whole-brain EEG patterns of sadness and disgust experiences. They found that amplitudes in the N2 and EPN allowed for an above-chance prediction whether disgust-related or sadness-related stimuli were presented.

A common denominator in research studying the neural basis of disgust is that it mostly relies on pictures from for example the International Affective Picture System (IAPS) or on movie clips to induce the aversive and repulsive response in participants (19, 20). Although it is conceivable that participants over-report successful emotion induction as a form of obsequiousness bias (21, 22), using pictures and videos for emotion induction has been shown to reliably trigger affective states (23). Nonetheless, there is still a marked difference in experiencing a threatening or disgusting stimulus only as a visual experience vs. experiencing said stimulus in real life, where the interaction with stimuli from the environment is usually involving multimodal sensory inputs. Furthermore, the emotional state is largely decoupled from behavioral and motivational states when the stimulus is presented on a computer screen. Emotions are multi-dimensional entailing not only an affective response but also physiological changes as well as behavioral tendencies (24). Experiencing fearful or disgusting stimuli would normally trigger a motivational state of avoidance to prevent harm (25) or contamination to oneself (26). These components of the emotional response are, however, unlikely to manifest both on the behavioral as well as neurophysiological level when participants passively perceive stimuli on a computer screen. Since this method of emotion induction is ubiquitous in neuroscience due to its advantage for event-related designs as well as constrained environments such as stationary EEGs or MRIs, the validity of these approaches to measure emotions as experienced in real-life remains is still debated.

To address this issue, there has been a call to increase ecological validity in affective neuroscience (27), which has already been implemented in several studies. For positive emotions, romantic partner interactions such as exchanging kisses or emotional and fond memories as well as compliments have been investigated using both EEG and fMRI (3, 28). Regarding negative emotions, fMRI studies have incorporated real human interactions such as insults by the experimenter to invoke feelings of anger in the participants (29, 30). A mobile EEG study has investigated the neural correlates of fear comparing a virtual plank-walking task eliciting strong and immersive fear reactions compared to the presentation of IAPS pictures in a within-subject design (31). Here, only the plank task was related to stronger relative frontal alpha power associated with stronger right-hemispheric activation (32). Recently, an EEG study by our group investigated frontal alpha asymmetries during a realistic stressful situation (33). We found a marked difference during acute stress on frontal and occipital electrodes with respect to asymmetry patterns whereas there was no difference in the following resting state measure.

Opposed to anger, fear and negative affect induced *via* stress, no study has, however, studied the neuronal correlates of disgust in ecologically valid settings. Aim of the present study is to fill this gap in the literature by investigating the influence of pictorial and naturalistic nauseating stimulus material as well as neutral control stimuli on alpha asymmetries as an indicator of emotional processing. Here, we specifically focused on frontal and occipital electrode sites in line with findings of Berretz et al. (33) who found effects in these regions during the induction of acute psychosocial stress. On the behavioral level, we predicted that nauseating stimuli would be perceived as more disgusting compared to neutral control stimuli. Furthermore, we expected that a naturalistic presentation of nauseating stimuli should lead to a stronger disgust response compared to a pictorial presentation. For the EEG data, we hypothesized that nauseating stimuli would lead to a stronger activation of the right frontal and occipital cortices reflected in higher left-hemispheric alpha power (34) compared to neutral control stimuli as negative and avoidance-related emotions are predominantly processed in the right hemisphere (35, 36). Finally, we hypothesized that naturalistic nauseating stimulus material would lead to a stronger increase in right-hemispheric activation compared to pictorial nauseating stimuli due to the multimodal and more realistic disgust experience.

## Materials and methods

### Participants

We gathered data from 56 participants (34 female, 22 male) at the Ruhr University Bochum, Germany. The sample size was based on previous results by Berretz et al. (33) who investigated the role of negative effect during acute psychosocial stress using a similar within-subjects protocol and the same analysis. To ensure that power was sufficient, we computed a sensitivity analysis at 80% power in a 2 × 2 ANOVA. With 56 participants, we would have been able to detect effect sizes of *d* = 0.38 which would correspond to small to medium effects. All participants were between 18 and 33 years old (mean = 23.9, SD = 3.7). 49 participants were right-handed and 7 were left-handed according to self-report. Participants were included if they had no history of previous or current mental or neurological disorders. Due to the nature of the stimulus material, we excluded participants with very high self-reported disgust sensitivity in a prescreening questionnaire using the German scale for disgust sensitivity [SADS (37)]. This was done to ensure that no participants would have an overwhelming visceral reaction to the stimulus material, which could compromise the participants’ well-being. The study was approved by the local ethics committee of the Faculty of Psychology at the Ruhr University Bochum. All participants were treated in accordance with the Declaration of Helsinki. Before the beginning of the testing session, participants were informed about the nature of the stimulus material in detail. All participants gave written informed consent and received a compensation of 20€ or course credit.

### Stimulus material

We used 10 different nauseating and corresponding neutral stimuli in the present study. The neutral stimuli were chosen to resemble the nauseating stimuli on both a visual and haptic level to exclude that changes in EEG signaling were due to low level perceptual rather than affective differences. For example, a non-inflated balloon (neutral) corresponded to a used condom (nauseating). Some stimuli were artificially created for the purpose of the study (see Supplementary Figures 1–10 for all stimuli). This information was however not given to the participants prior to the study to induce a strong feeling of disgust.

1. Disgust: Sheep eyes/Neutral: Candy eyeballs
2. Disgust: Sheep lung/Neutral: Sponge
3. Disgust: Sheep intestine/Neutral: Plastic tubes
4. Disgust: Used condom (filled with soap)/Neutral: Airless balloon
5. Disgust: (Fake) vomit/Neutral: Oatmeal
6. Disgust: (Fake) blood/Neutral: Tomato sauce
7. Disgust: Used tampon (soaked in venous blood)/Neutral: Unused tampon
8. Disgust: Rotten sandwich and yogurt/Neutral: Fresh sandwich and yogurt
9. Disgust: Hair pulled from the shower drain/Neutral: cord
10. Disgust: Dead mealworms/Neutral: Candy worms

These stimuli were chosen to cover a large variety of disgust eliciting stimuli that can be encountered in everyday life. Core disgust was elicited by vomit and mealworms, blood-injury disgust was elicited by sheep lungs, eyes, and intestine as well as blood. Interpersonal and sexual disgust of diseased individuals and inappropriate sexual contact were evoked by the used condom, used hygiene products and hair pulled from the drain.

The stimuli were kept in a refrigerator at four degrees Celsius in the experimental room to maintain their status over duration of the study. Stimuli were exchanged for new ones in case of notable differences in integrity, smell, or rottenness. Glass containers for keeping and presentation of some stimuli (see Supplementary material) were acquired at IKEA (Korken, 1l) and sealed with glue and parafilm. All other stimuli were kept and presented in air sealed plastic bags used for vacuum sealing food. Participants were able to see and handle all naturalistic stimuli but were not able to smell or directly touch the stimuli to avoid contamination with potential germs that could compromise participants’ health.

### Procedure

**FIGURE 1:**
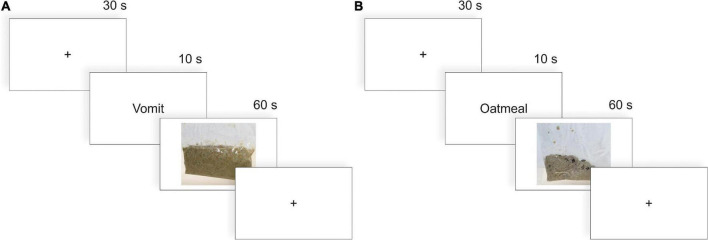
Procedure of each individual trial in the **(A)** nauseating and **(B)** neutral condition. Before each trial, the participants were asked to fixate on a fixation cross for 30 s. Then, the next item to be presented was shown for 10 s on the screen. In a pictorial block, this item was then presented on the screen for 60 s (illustrated here). In a naturalistic block, the experimenter brought this item to the participant and took it away as soon as the 60 s ended.

After giving consent, participants were outfitted with a 64-channel EEG cap and underwent a 5-min resting-state measure to ensure high data quality from all channels. Afterward, participants were presented with four different blocks of stimuli: 10 naturalistic disgusting stimuli, 10 corresponding naturalistic neutral stimuli as well as 10 pictures of disgusting stimuli, and 10 pictures of neutral stimuli. The order of blocks as well as the order of stimuli within each block was counterbalanced across participants to minimize any sequential effects in the data. The naturalistic stimuli were presented in either a plastic bag or a glass jar. The pictorial stimuli were photographs of their real counterparts to match the visual sensation. Each trial started with a fixation cross in the middle of the screen for 30 s after which a word corresponding to the following stimulus was shown for 10 s. In naturalistic conditions, an experimenter would bring the stimuli to the participants during this period and remove the previous stimulus. Stimuli were placed on the desk between the head rest and the computer monitor. Stimuli were presented for 60 s and removed from the room by the experimenter or replaced with a fixation cross on the screen after this time period (see Figure 1 for the trial procedure). During the presentation of the stimuli, the experimenter left the room and closed the door so that participants were alone during the examination of naturalistic and pictorial stimuli. Participants were instructed to inspect all stimuli intently and to touch the naturalistic stimulus material. After each block of stimulus type, participants were asked to rate how disgusting each stimulus was and how uncomfortable they imagined handling each stimulus would be on a 0 (not disgusting/uncomfortable) to 10 (extremely disgusting/uncomfortable) scale. Triggering stimulus on- and offset in the EEG data in the pictorial conditions was automatic. In the naturalistic conditions, a second experimenter manually set triggers for stimulus onset waiting for the exact moment, the naturalistic stimuli were placed in front of the participants. Stimulus offset was set automatically after 60 s.

### EEG recording and analysis

We recorded EEG data with a 64 Ag–Ag Cl electrode system (actiCAP ControlBox and QuickAmp 72, Brain Products GmbH, Gilching, Germany). Electrodes were positioned according to the international 10–20 system (FCz, FP1, FP2, F7, F3, F4, F8, FC5, FC1, FC2, FC6, T7, C3, Cz, C4, T8, TP9, CP5, CP1, CP2, CP6, TP10, P7, P3, Pz, P4, P8, PO9, O1,Oz, O2, PO10, AF7, AF3, AF4, AF8, F5, F1, F2, F6, FT9, FT7, FC3, FC4, FT8, FT10,C5, C1, C2, C6, TP7, CP3, CPz, CP4, TP8, P5, P1, P2, P6, PO7, PO3, POz, PO4, PO8). The sampling rate during recording was 1 kHz. The reference electrode was located at the FCz position. Data analysis was performed offline using the Brain Vision Analyzer software (Brain Products GmbH). In a first step, data were down-sampled to 500 Hz and visually inspected for motion artifacts as well as faulty channels. Subsequently, data were filtered with a low cutoff filter of 0.5 Hz and a high cutoff filter of 30 Hz, and an additional notch filter of 50 Hz. After that, we applied a semiautomatic independent component analysis (ICA) with Infomax rotation to eliminate reoccurring artifacts like pulse, blinks, and eye movements from the data. Following this, data were epoched into 60-s segments corresponding to each stimulus. Data were baseline corrected across the whole segment length before cutting each segment into epochs of 1,024 ms. Lastly, we performed an automatic artifact rejection, which excluded segments with voltage steps of more than 50 μV/ms, a maximum value difference of outside the range of 200 μV within a 200 ms interval or amplitudes below 0.1 μV. For the naturalistic disgusting and the naturalistic neutral conditions, 2.6% of segments were rejected each. For the pictorial disgusting and the pictorial neutral condition, 1 and 1.7% of segments were rejected, respectively.

### Statistical analysis

For analysis, we focused on two primary outcomes measures. First, we looked at behavioral responses to the questionnaires as these are the most informative to assess the participants’ individual emotional responses to the stimuli. As a second outcome measure, we focused on alpha asymmetries as these have been reliably shown to be associated with emotional processing in humans [for review, see Reznik and Allen (32)].

To analyze the behavioral data, we performed a repeated-measures ANOVA with the factor condition (pictorial vs. naturalistic) and the factor emotion (neutral vs. nauseating) on the ratings of disgust and imagined discomfort. For the analysis of the EEG data, we focused on asymmetries in the alpha frequency band (8–13 Hz) at the electrode pairs F3/4, F5/6, F7/8, and O1/2. Electrodes were chosen identically to the study of Berretz et al. (33) as both frontal and occipital electrodes showed changes in asymmetry patterns during acute psychosocial stress. Asymmetries on these frontal electrodes have been suggested to be involved in emotional regulation (38, 39). The occipital electrodes were chosen as they map onto the alpha frequency generators in the brain (34). We extracted alpha power as an average across all stimulus presentations per electrode and then calculated asymmetry indices (AIs) for the alpha power for each electrode pair following the formula by Reznik and Allen (32):


AI=ln⁢(right)-ln⁢(left)

Asymmetry indices are most easily interpreted as follows: a relatively smaller AI is indicative of relatively higher alpha power in the left hemisphere. Since alpha power is negatively correlated with activation (34), this is indicative of stronger right-hemispheric activation. A relatively higher AI is therefore indicative of stronger relative left-hemispheric activation.

We performed one repeated-measures ANOVA on the AIs with the factor condition (pictorial vs. naturalistic), and the factor emotion (neutral vs. nauseating) for each electrode pair (F3/4, F5/6, F7/8, O1/2). All *post-hoc* tests were Bonferroni corrected. To assess the evidence not only for the alternative but also the null hypothesis, we complemented all analyses with their Bayesian analogues to obtain Bayes factors (BFs). The BF represents the amount of evidence for the null or alternative. For example, a BF of 2 indicates that there is twice as much evidence for the alternative compared to the null hypothesis. While BFs provide continuous measures of evidence, common thresholds to mark moderate evidence are a BF > 3 for the alternative hypothesis and a BF < 1/3 for the null hypothesis (40). The BF_*incl*_ will be used for the results of ANOVAs and the BF_10_ will be used for relevant *post-hoc* tests. Bayesian analyses were computed in JASP (v 16.4.0) using default priors.

## Results

### Behavioral results

**FIGURE 2:**
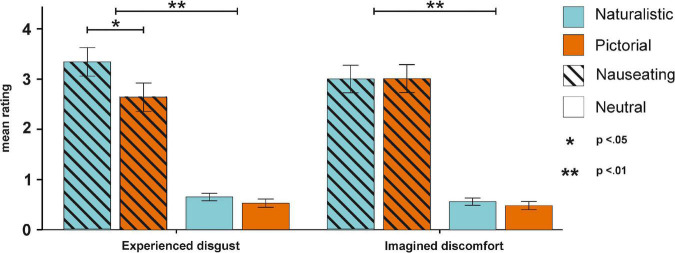
Behavioral results for the experienced disgust and imagined discomfort ratings. Experienced disgust was more pronounced for nauseating stimuli, especially in naturalistic conditions. Imagined discomfort only differed between nauseating and neutral stimuli.

The ANOVA on mean disgust ratings (see Supplementary Table 1 for descriptive data) with the factors condition and emotion revealed a significant main effect of emotion [*F*_(1,55)_ = 108.93, *p* < 0.001, η_*p*_^2^ = 0.66, BF_*incl*_ > 100; Supplementary Table 2] with higher disgust ratings for nauseating compared to neutral stimuli. Here, both nauseating naturalistic (*p* < 0.001, BF_10_ > 100) and pictorial stimuli (*p* < 0.001, BF_10_ > 100) were perceived as more disgusting compared to their neutral counterparts. Moreover, the interaction between condition and emotion also reached significance [*F*_(1,55)_ = 5.13, *p* = 0.027, η_*p*_^2^ = 0.09, BF_*incl*_ = 9.26]. Here, we found higher disgust ratings for nauseating naturalistic compared to nauseating pictorial stimuli (*p* = 0.005, BF_10_ = 6.80). The ANOVA on mean imagined discomfort ratings (see Supplementary Table 1 for descriptive data) with the factors condition and emotion resulted only in a significant main effect of emotion [*F*_(1,55)_ = 112.23, *p* < 0.001, η_*p*_^2^ = 0.67, BF_*incl*_ > 100; Supplementary Table 3] with higher discomfort ratings for nauseating compared to neutral stimuli (see Figure 2). Again, both nauseating naturalistic (*p* < 0.001, BF_10_ > 100) and pictorial stimuli (*p* < 0.001, BF_10_ > 100) were associated with higher discomfort relative to their neutral counterpart.

### EEG results

**FIGURE 3:**
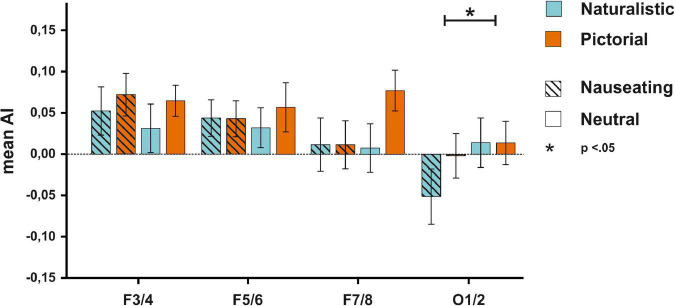
EEG results for the F3/4, F5/6, F7/8, and O1/2 electrodes for all participants. No significant differences in activity patterns were observed on frontal electrodes between any experimental block. For the O1/2 electrode, smaller AIs could be detected for nauseating compared to neutral stimuli.

The ANOVA on AIs at the F3/4 electrode pair (see Supplementary Table 4 for descriptive data) with the factors condition and emotions resulted in no significant main effect of condition [*F*_(1,55)_ = 3.72, *p* = 0.059, η_*p*_^2^ = 0.06, BF_*incl*_ = 0.52], nor a significant main effect of emotion [*F*_(1,55)_ = 1.00, *p* = 0.321, η_*p*_^2^ = 0.02, BF_*incl*_ = 0.20]. The interaction between both factors also failed to reach significance [*F*_(1,55)_ = 0.25, *p* = *0.622*, η_*p*_^2^ < 0.01, BF_*incl*_ = 0.08]. Similarly, the ANOVA on AIs at the F5/6 electrode pair with the same factors showed no main effect of condition [*F*_(1,55)_ = 0.30, *p* = 0.584, η_*p*_^2^ < 0.01, BF_*incl*_ = 0.16], no main effect of emotion [*F*_(1,55)_ = 0.01, *p* = 0.944, η_*p*_^2^ < 0.01, BF_*incl*_ = 0.12] and no significant interaction between both factors [*F*_(1,55)_ = 0.59, *p* = 0.448, η_*p*_^2^ < 0.01, BF_*incl*_ = 0.03]. The ANOVA on AIs at the F7/8 electrode pair showed no significant main effect of condition [*F*_(1,55)_ = 1.94, *p* = 0.170, η_*p*_^2^ = 0.03, BF_*incl*_ = 0.37], nor a significant main effect of emotion [*F*_(1,55)_ = 2.45, *p* = 0.123, η_*p*_^2^ = 0.04, BF_*incl*_ = 0.36], and no significant interaction between condition and emotion [*F*_(1,55)_ = 2.76, *p* = 0.102, η_*p*_^2^ = 0.05, BF_*incl*_ = 0.35]. While the ANOVA on AIs at the O1/2 electrode pair also showed no significant main effect of condition [*F*_(1,55)_ = 1.66, *p* = 0.203, η_*p*_^2^ = 0.03, BF_*incl*_ = 0.35], there was a significant main effect of emotion [*F*_(1,55)_ = 5.73, *p* = 0.020, η_*p*_^2^ = 0.09, BF_*incl*_ = 1.22] with smaller AIs in the disgust compared to the neutral condition suggesting stronger right-hemispheric activation. The interaction between the factors condition and emotion was not significant [*F*_(1,55)_ = 2.41, *p* = 0.126, η_*p*_^2^ = 0.04, BF_*incl*_ = 0.46; see Figure 3].

A notable result in the behavioral ratings was that a subset of participants was not disgusted by any stimuli at all (average disgust score for naturalistic nauseating stimuli ≤ 2; *N* = 18). Since we pre-selected our participants before the study to not be overly sensitive to disgust, a possible side effect could have been that the sample was skewed toward people who are not easily disgusted. We decided to exclude these participants in an exploratory analysis to only evaluate the neuronal signals of participants with an overall self-report of a disgusting experience in the most disgusting condition. Removing these 18 participants resulted in a sample of 38 participants (24 women, mean age = 23.1 years).

**FIGURE 4:**
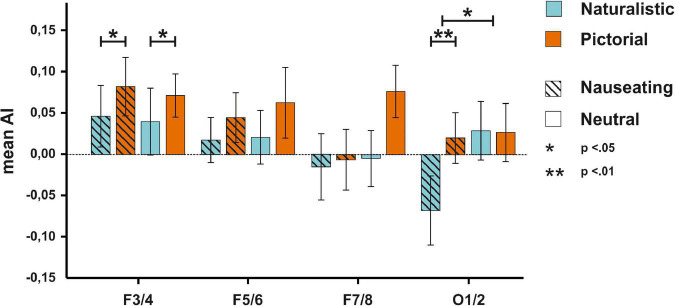
EEG results for the F3/4, F5/6, F7/8, and O1/2 electrodes for the participants with a disgust response. On the F3/4 electrode, pictorial stimuli were associated with higher AIs. On the O1/2 electrode, disgusting stimuli were related to smaller AIs compared to neutral stimuli. Furthermore, naturalistic disgust stimuli elicited more negative AIs compared to pictorial disgust stimuli.

**FIGURE 5:**
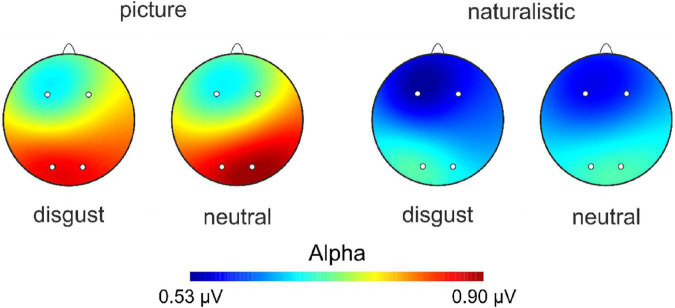
Scalp topography for alpha power across the four experimental conditions.

We thus repeated the analyses mentioned above with participants that displayed an emotional reaction to the stimulus material. The ANOVA on AIs at the F3/4 electrode revealed a significant main effect of condition [*F*_(1,37)_ = 5.46, *p* = 0.025, η_*p*_^2^ = 0.13; see Supplementary Table 5 for descriptive data] with smaller AIs in the naturalistic condition suggesting stronger right-hemispheric activation when naturalistic stimuli were presented. Of note is that the Bayesian ANOVA tentatively suggested absence of evidence (BF_*incl*_ = 0.73). No other main effect or interaction was significant (*p*s > 0.635, all BF_*incl*_ < 0.18). The ANOVA on AIs at the F5/6 electrode pair showed no significant main effect of condition [*F*_(1,37)_ = 1.50, *p* = 0.229, η_*p*_^2^ = 0.04, BF_*incl*_ = 0.31], nor a significant main effect of emotion [*F*_(1,37)_ = 0.21, *p* = 0.652, η_*p*_^2^ < 0.01, BF_*incl*_ = 0.16] and no significant interaction between condition and emotion [*F*_(1,37)_ = 0.10, *p* = 0.750, η_*p*_^2^ < 0.01, BF_*incl*_ = 0.05]. The ANOVA on AIs at the F7/8 electrode pair revealed no significant main effect of condition [*F*_(1,37)_ = 1.98, *p* = 0.168, η_*p*_^2^ = 0.02, BF_*incl*_ = 0.42], nor a significant main effect of emotion [*F*_(1,37)_ = 2.94, *p* = 0.095, η_*p*_^2^ = 0.07, BF_*incl*_ = 0.50], and no significant interaction between condition and emotion [*F*_(1,37)_ = 1.79, *p* = 0.189, η_*p*_^2^ = 0.05, BF_*incl*_ = 0.30]. Finally, the ANOVA on AIs at the O1/2 electrode pair with the same factors demonstrated a significant main effect of emotion [*F*_(1,37)_ = 5.53, *p* = 0.024, η_*p*_^2^ = 0.17, BF_*incl*_ = 3.49] with smaller AIs in response to nauseating stimuli. Furthermore, the interaction between condition and emotion also reached significance [*F*_(1,37)_ = 5.03, *p* = 0.031, η_*p*_^2^ = 0.12, BF_*incl*_ = 5.24]. Here, we found smaller AIs in response to naturalistic nauseating stimuli compared to pictorial nauseating and neutral stimuli (*p* = 0.002, BF_10_ = 17.09, see Figure 4) suggesting that naturalistic nauseating stimuli evoked stronger right-hemispheric activation compared to a pictorial presentation. A scalp view of the result pattern is shown in Figure 5.

## Discussion

In the present study, we investigated whether pictorial and naturalistic nauseating stimuli elicit differential responses in terms of perceived disgust and in the EEG signal. To this end, participants were presented with naturalistic and pictorial nauseating stimuli. Each stimulus also had a neutral counterpart similar in haptic and visual features to control for sensory differences in the objects. On the behavioral level, participants rated the nauseating stimuli as more disgusting compared to the neutral stimuli. Furthermore, naturalistic nauseating stimuli were perceived as more disgusting than their pictorial counterparts. On the neurophysiological level, we found that naturalistic nauseating stimuli lead to a stronger relative activation of the right hemisphere compared to pictorial stimuli at the alpha oscillation generators at occipital sites. When we only analyzed individuals with a disgust response to the nauseating stimuli, we furthermore found an interaction between the valence of the stimuli and their presentation type. Here, naturalistic nauseating stimuli were associated with stronger relative right-hemispheric activation compared to pictorial nauseating stimuli. No such effect was observed for neutral stimuli.

In accordance with our hypotheses, participants perceived nauseating stimuli as significantly more disgusting than control stimuli, which were not evaluated as disgusting at all. This indicates a reasonable choice of stimulus material as neutral stimuli were rated as neutral while still providing similar visual and haptic features. Among the nauseating stimuli, naturalistic ones were perceived as significantly more disgusting than pictures of the stimulus material. This suggests that real-life stimuli elicit a stronger emotional reaction than merely looking at pictures of the same content. Previous studies have demonstrated that pictures are generally inferior to other methods of emotional induction. For example, movie clips show a stronger effect for emotion induction compared to pictures (41, 42). Moreover, emotion regulation in response to pictures is easier for participants than in response to movies (43, 44) indicating that movies have a longer lasting and more severe impact on acute affect. Recent studies also made use of virtual reality (VR) to increase the effectiveness of emotion induction (45). In a study by Li et al. (46), the authors utilized an interactive VR environment for induction of emotions. The authors found that VR not only increased the emotional arousal of the participants but also only VR stimulus material induced asymmetries in the alpha power band. This suggests that the more naturalistic and engaging the stimulus material is, the better it is suited for the induction of affective states.

Future research on emotion processing and regulation may benefit from incorporating naturalistic stimuli. In the case of disgust, studies examining the visual exploration of emotion inducing stimuli have typically used pictorial stimuli (47). In addition, visual inspection and behavioral approach and avoidance tendencies in response to naturalistic stimuli could strengthen the interpretation of results. Especially the evaluation of emotion regulation strategies for disgust related disorders like Obsessive Compulsive Disorder (48) may profit from the use of naturalistic stimuli as patients encounter these stimuli outside of the therapy setting.

An interesting finding was observed in the difference between the perceived disgust and imagined discomfort scale that the participants had to report following their engagement with the stimulus material. While participants displayed increased experienced disgust in response to naturalistic compared to pictorial nauseating stimuli, there was no significant difference in imagined discomfort between naturalistic and pictorial stimuli. Since they were specifically asked to picture themselves interacting with the stimuli, this difference in results could be explained by the fact that participants were similarly good in imagining an interaction with the stimulus material regardless of mode of presentation thus leading to no difference in imagined discomfort (49).

On the neurophysiological level, we could partly confirm our hypothesis that the processing of disgusting stimuli is associated with a stronger recruitment of the right hemisphere as we found stronger right-hemispheric activation in response to nauseating compared to neutral stimuli at the O1/2 electrode pair. This is in line with previous work proposing that the right hemisphere is dominant for eliciting and processing negative and withdrawal-related emotions (50, 51). Contrasting our prediction and the behavioral findings however, there was no difference between the presentation of naturalistic and pictorial stimuli when the whole sample was investigated. This discrepancy could be explained by differences in signal-to-noise ratio in the data. Participants without any reported disgust response were likely to contribute noise, as they did not experience any negative affect. Thus, although the power was effectively reduced by decreasing the sample size, we could find an interaction effect in a sub-sample of individuals who reported an overall disgust experience throughout the task. As hypothesized, we found stronger relative right-hemispheric activation in response to naturalistic compared to pictorial nauseating stimuli indicating that the method of presentation not only affects behavioral but also cerebral responses associated with avoidance and negative affect. These findings suggest that naturalistic stimuli are superior in emotion research and that pictures are suboptimal to induce affective states in line with other studies that failed to find changes in asymmetry using pictorial stimuli (31, 52–54). It could be argued that these results could be attributed to higher arousal resulting from the presentation of naturalistic stimuli. Indeed, higher arousal has been associated with lower alpha power (55). However, alpha asymmetries are a relative measure, comparing left-hemispheric to right-hemispheric EEG activity. This suggests that the overall level of arousal and thus activation is irrelevant unless arousal states activate one hemisphere more strongly than the other. Since there is a wide body of evidence supporting the notion that differences in emotional valence elicit asymmetrical activation patterns, but evidence for lateralization of arousal is sparse (56), we do not believe that arousal played a major role in our observed results.

The usefulness of naturalistic stimulus material compared to pictures may be especially pronounced when investigating disgust specifically. Pictorial stimuli can be viewed indefinitely without any real risk and thus do not require a fast reaction. Previous studies have shown that pictorial disgust stimuli are viewed longer than fear-inducing stimuli while not being processed better (47, 57). Pictures of disgusting stimuli could trigger a form of morbid curiosity, paradoxically leading to the seeking out of these pictures (58). Naturalistic stimuli on the other hand offer the direct threat of contamination necessitating a quick and decisive reaction. In an experimental setting without the possibility to avoid the stimulus material, this could lead to the induction of stronger emotions.

A similar effect could be induced by selecting maximally negatively rated pictures. However, this may run into a sort of ceiling effect, as the maximal disgust strength induced by pictures is still likely lower than the disgust induced by disgusting naturalistic stimuli. Nevertheless, using pictures for emotion induction is still useful, especially when investigating sensitive populations like strongly-affected patients or children since outcomes like emotion regulation can also be investigated with pictures (59).

Interestingly, only for this sub-sample of participants, we found an effect on frontal electrodes. Although we predicted the same pattern for frontal and occipital sites, no difference between the presentation of nauseating and neutral stimuli was observed at frontal electrode sites. Rather, frontal electrodes showed differences in asymmetry between the presentation of naturalistic and pictorial stimuli. A possible explanation for these results could relate to frontal alpha asymmetry being associated with the processing of salience as shown for example in studies using salient erotic stimuli (60, 61). The differential pattern between frontal and occipital electrodes resembles findings observed by Berretz et al. (33) during the measurement of acute psychosocial stress as increases in right hemispheric activation were also selective for occipital sites. Frontal sites however showed signal changes associated with emotional challenge in line with the capability model of emotions (62, 63). These findings suggest that asymmetry patterns are not uniform across the scalp as observed in several other EEG studies studying both negative and positive affect [e.g., (28, 64)]. It could be speculated that affective patterns are most strongly represented at the origin of alpha power oscillations as alpha rhythms are generated at the occipital cortex (65). Frontal alpha asymmetries might however represent not exclusively affect, but also salience or signals of emotional challenge as well as emotional regulation (38, 39) and are thus more complex in nature. Since our significant result on frontal electrodes was not supported by a Bayes factor in favor of the alternative hypothesis, this result should be treated with caution, however.

### Limitations and future directions

As already pointed out, a possible limitation of the current study can be found in the selection of participants. To ensure that no participants had a severe aversive reaction toward the stimulus material, we specifically recruited participants that reported a high tolerance for disgust during prescreening. This could have obscured more nuanced effects and overall limited the generalizability of our results to the population at large. Moreover, applying naturalistic stimuli in research might come with its own caveats. Using real fear or sadness-inducing stimulus material may constitute a serious burden to participants and might not always be ethically achievable. This may be specifically problematic when working with vulnerable populations like children or patient cohorts. Nevertheless, we believe that introducing more naturalistic stimuli into neuroscientific research can help understand inconsistencies in results between studies and lead to more valid interpretations of the literature, especially in the heterogeneous field of emotional lateralization.

Another potential limitation pertains to the differences in modality between conditions. In the naturalistic task, there was both haptic and visual input whereas the pictorial condition was exclusively visual. Thus, it cannot be definitively ruled out that our obtained results were due to haptic differences. As pointed out, however, the computation of asymmetry indices should remove any bilateral activation patterns. Since the participants explored the objects with both hands, haptic input should have been the same between both conditions. We thus do not believe that modality was of major influence.

Finally, our experimental design unfortunately does not allow us to look at ERP components that had been previously associated with the processing of disgust (e.g., N1 or EPN). The computation of ERPs requires block designs with millisecond accuracy trigger events. This was not possible for the naturalistic presentation since the onset of the trial was manually triggered by the experimenters. Furthermore, the number of trials was too low to look at ERPs reliably.

## Conclusion

In summary, we found that naturalistic nauseating stimuli are more effective to induce disgust on the subjective level. These results were supported by stronger right-hemispheric occipital brain activity. Future studies should therefore aim to increase the ecological validity of the emotional situation and the stimulus material to increase the odds of inducing realistic affective states in laboratory settings.

## Data availability statement

The raw data supporting the conclusions of this article will be made available by the authors, without undue reservation.

## Ethics statement

The studies involving human participants were reviewed and approved by Local Ethics Committee, Ruhr University Bochum. The patients/participants provided their written informed consent to participate in this study.

## Author contributions

GB and JP conceptualized and designed the study and wrote the manuscript. CD and EL collected the EEG data. GB processed and analyzed the data. JP consulted on data analysis. All authors contributed to the article and approved the submitted version.

## References

[1] Rozin P Haidt J McCauley CR. Disgust. 3rd ed. In: Lewis M Haviland-Jones JM Barret LF editors. *Handbook of emotions.* New York, NY: Guilford Press (2008). p. 757–76.

[2] Darwin C Prodger P. *The expression of the emotions in man and animals.* Oxford: Oxford University Press (1998).

[3] Eckstein M Stoessel G Gerchen MF Bilek E Kirsch P Ditzen B. Neural responses to instructed positive couple interaction: an fMRI study on compliment sharing. *BioRxiv.* [Preprint]. (2022). 10.1101/2022.06.15.496238 PMC9976881 36852857

[4] Lindquist KA Barrett LF. A functional architecture of the human brain: emerging insights from the science of emotion. *Trends Cogn Sci.* (2012) 16:533–40. 10.1016/j.tics.2012.09.005 23036719 PMC3482298

[5] Lindquist KA Wager TD Kober H Bliss-Moreau E Barrett LF. The brain basis of emotion: a meta-analytic review. *Behav Brain Sci.* (2012) 35:121–43. 10.1017/S0140525X11000446 22617651 PMC4329228

[6] Curtis V Aunger R Rabie T. Evidence that disgust evolved to protect from risk of disease. *Proc Biol Sci.* (2004) 271(suppl. 4):S131–3. 10.1098/rsbl.2003.0144 15252963 PMC1810028

[7] Oaten M Stevenson RJ Case TI. Disgust as a disease-avoidance mechanism. *Psychol Bull.* (2009) 135:303. 10.1037/a0014823 19254082

[8] Chapman HA Anderson AK. Understanding disgust. *Ann N Y Acad Sci.* (2012) 1251:62–76. 10.1111/j.1749-6632.2011.06369.x 22256964

[9] Wright P He G Shapira NA Goodman WK Liu Y. Disgust and the insula: fMRI responses to pictures of mutilation and contamination. *Neuroreport.* (2004) 15:2347–51. 15640753 10.1097/00001756-200410250-00009

[10] Yan H Wang Y Tian J Liu Y. Effective connectivity of neural pathways underlying disgust by multivariate Granger causality analysis. In: Weaver JB Molthen RC editors. *SPIE proceedings, medical imaging 2011: biomedical applications in molecular, structural, and functional imaging.* Bellingham: SPIE (2011). p. 796504. 10.1117/12.877642

[11] Schienle A Stark R Walter B Blecker C Ott U Kirsch P The insula is not specifically involved in disgust processing: an fMRI study. *Neuroreport.* (2002) 13:2023–6. 12438918 10.1097/00001756-200211150-00006

[12] Barrett LF Mesquita B Ochsner KN Gross JJ. The experience of emotion. *Annu Rev Psychol.* (2007) 58:373–403. 10.1146/annurev.psych.58.110405.085709 17002554 PMC1934613

[13] Gan X Zhou X Li J Jiao G Jiang X Biswal B Common and distinct neurofunctional representations of core and social disgust in the brain: an ALE meta-analysis and MACM characterization. *BioRxiv.* [Preprint]. (2021). 10.1101/2021.09.07.459241 35122784

[14] Schaich Borg J Lieberman D Kiehl KA. Infection, incest, and iniquity: investigating the neural correlates of disgust and morality. *J Cogn Neurosci.* (2008) 20:1529–46. 10.1162/jocn.2008.20109 18345982 PMC3969035

[15] Li Z Li Y Li X Zou F Wang Y Wu X The spontaneous brain activity of disgust: perspective from resting state fMRI and resting state EEG. *Behav Brain Res.* (2021) 403:113135. 10.1016/j.bbr.2021.113135 33476686

[16] Carretié L Ruiz-Padial E López-Martín S Albert J. Decomposing unpleasantness: differential exogenous attention to disgusting and fearful stimuli. *Biol Psychol.* (2011) 86:247–53. 10.1016/j.biopsycho.2010.12.005 21184798

[17] Wheaton MG Holman A Rabinak CA MacNamara A Proudfit GH Phan KL. Danger and disease: electrocortical responses to threat-and disgust-eliciting images. *Int J Psychophysiol.* (2013) 90:235–9. 10.1016/j.ijpsycho.2013.08.001 23938878

[18] Revers H van Deun K Strijbosch W Vroomen J Bastiaansen M. Decoding the neural responses to experiencing disgust and sadness. *Brain Res.* (2022) 1793:148034. 10.1016/j.brainres.2022.148034 35908590

[19] Courtney CG Dawson ME Schell AM Iyer A Parsons TD. Better than the real thing: eliciting fear with moving and static computer-generated stimuli. *Int J Psychophysiol.* (2010) 78:107–14. 10.1016/j.ijpsycho.2010.06.028 20600370

[20] Lang PJ. *International affective picture system (IAPS): Affective ratings of pictures and instruction manual*, Technical Report A-8. Gainesville, FL: University of Florida (2005).

[21] Delgado-Rodríguez M Llorca J. Bias. *J Epidemiol Community Health.* (2004) 58:635–41. 10.1136/jech.2003.008466 15252064 PMC1732856

[22] Sackett D. Bias in analytic research. *J Chronic Dis.* (1979) 32:51–63. 447779 10.1016/0021-9681(79)90012-2

[23] Horvat M Kukolja D Ivanec D. Comparing affective responses to standardized pictures and videos: a study report. *MIPRO, 2015 proceedings of the 38th international convention.* Piscataway, NJ: IEEE (2015). p. 1394–8.

[24] Güntürkün O. *Biologische psychologie.* Göttingen: Hogrefe Verlag (2019).

[25] Wong AHK Pittig A. Avoiding a feared stimulus: modelling costly avoidance of learnt fear in a sensory preconditioning paradigm. *Biol Psychol.* (2022) 168:108249. 10.1016/j.biopsycho.2021.108249 34973369

[26] Davey GCL. Disgust: the disease-avoidance emotion and its dysfunctions. *Philos Trans R Soc Lond B Biol Sci.* (2011) 366:3453–65. 10.1098/rstb.2011.0039 22042921 PMC3189352

[27] Ocklenburg S Berretz G Packheiser J Friedrich P. Laterality 2020: entering the next decade. *Laterality.* (2021) 26:265–97. 32787546 10.1080/1357650X.2020.1804396

[28] Packheiser J Berretz G Rook N Bahr C Schockenhoff L Güntürkün O Investigating real-life emotions in romantic couples: a mobile EEG study. *Sci Rep.* (2021) 11:1142. 10.1038/s41598-020-80590-w 33441947 PMC7806608

[29] Denson TF Pedersen WC Ronquillo J Nandy AS. The angry brain: neural correlates of anger, angry rumination, and aggressive personality. *J Cogn Neurosci.* (2009) 21:734–44. 18578600 10.1162/jocn.2009.21051

[30] Gilam G Lin T Raz G Azrielant S Fruchter E Ariely D Neural substrates underlying the tendency to accept anger-infused ultimatum offers during dynamic social interactions. *NeuroImage.* (2015) 120:400–11. 10.1016/j.neuroimage.2015.07.003 26166623

[31] El Basbasse Y Packheiser J Peterburs J Maymon C Güntürkün O Grimshaw G Walk the plank! using mobile EEG to investigate emotional lateralization of immersive fear in virtual reality. *boiRxiv.* [Preprint]. (2022). 10.1101/2022.08.30.505699 PMC10230188 37266038

[32] Reznik SJ Allen JJB. Frontal asymmetry as a mediator and moderator of emotion: an updated review. *Psychophysiology.* (2018) 55:e12965. 10.1111/psyp.12965 28776710

[33] Berretz G Packheiser J Wolf OT Ocklenburg S. Acute stress increases left hemispheric activity measured via changes in frontal alpha asymmetries. *Iscience.* (2022) 25:103841. 10.1016/j.isci.2022.103841 35198894 PMC8850739

[34] Bazanova OM Vernon D. Interpreting EEG alpha activity. *Neurosci Biobehav Rev.* (2014) 44:94–110. 10.1016/j.neubiorev.2013.05.007 23701947

[35] Gainotti G. A historical review of investigations on laterality of emotions in the human brain. *J Hist Neurosci.* (2019) 28:23–41. 10.1080/0964704X.2018.1524683 30475661

[36] Gainotti G. Emotions and the right hemisphere: editorial. *Brain Sci.* (2021) 11:1579. 10.3390/brainsci11121579 34942881 PMC8699496

[37] Schienle A Dietmaier G Ille R Leutgeb V. Eine skala zur erfassung der ekelsensitivität (SEE). *Zeitschrift Für Klinische Psychol Und Psychother.* (2010) 39:80–6. 10.1026/1616-3443/a000016

[38] Choi D Sekiya T Minote N Watanuki S. Relative left frontal activity in reappraisal and suppression of negative emotion: evidence from frontal alpha asymmetry (FAA). *Int J Psychophysiol.* (2016) 109:37–44. 10.1016/j.ijpsycho.2016.09.018 27693504

[39] Zhang J Hua Y Xiu L Oei TP Hu P. Resting state frontal alpha asymmetry predicts emotion regulation difficulties in impulse control. *Personal Individ Diff.* (2020) 159:109870.

[40] Lee MD Wagenmakers EJ. *Bayesian cognitive modeling: a practical course.* Cambridge: Cambridge university press (2014). 10.1017/CBO9781139087759

[41] Ellard KK Farchione TJ Barlow DH. Relative effectiveness of emotion induction procedures and the role of personal relevance in a clinical sample: a comparison of film, images, and music. *J Psychopathol Behav Assess.* (2012) 34:232–43. 10.1007/s10862-011-9271-4 34611377 PMC8489569

[42] Julien E Over R. Male sexual arousal across five modes of erotic stimulation. *Arch Sex Behav.* (1988) 17:131–43. 10.1007/BF01542663 2456050

[43] Dhaka S Kashyap N. Explicit emotion regulation: comparing emotion inducing stimuli. *Psychol Thought.* (2017) 10:303–14. 10.5964/psyct.v10i2.240 33680184

[44] Webb TL Miles E Sheeran P. Dealing with feeling: a meta-analysis of the effectiveness of strategies derived from the process model of emotion regulation. *Psychol Bull.* (2012) 138:775–808. 10.1037/a0027600 22582737

[45] Meuleman B Rudrauf D. Induction and profiling of strong multi-componential emotions in virtual reality. *IEEE Trans Affect Comput.* (2018) 12:189–202.

[46] Li M Pan J Gao Y Shen Y Luo F Dai J Neurophysiological and subjective analysis of VR emotion induction paradigm. *IEEE Trans Vis Comput Graph.* (2022) 28:3832–42. 10.1109/TVCG.2022.3203099 36049001

[47] Fink-Lamotte J Svensson F Schmitz J Exner C. Are you looking or looking away? Visual exploration and avoidance of disgust-and fear-stimuli: an eye-tracking study. *Emotion.* (2021) 22:1909–18. 34582239 10.1037/emo0000993

[48] Fink J Pflugradt E Stierle C Exner C. Changing disgust through imagery rescripting and cognitive reappraisal in contamination-based obsessive-compulsive disorder. *J Anxiety Disord.* (2018) 54:36–48. 10.1016/j.janxdis.2018.01.002 29421371

[49] Burleigh L Jiang X Greening SG. Fear in the theater of the mind: differential fear conditioning with imagined stimuli. *Psychol Sci.* (2022) 33:1423–39. 10.1177/09567976221086513 35895306 PMC13021123

[50] Harmon-Jones E Gable PA Peterson CK. The role of asymmetric frontal cortical activity in emotion-related phenomena: a review and update. *Biol Psychol.* (2010) 84:451–62. 10.1016/j.biopsycho.2009.08.010 19733618

[51] Palmiero M Piccardi L. Frontal EEG asymmetry of mood: a mini-review. *Front Behav Neurosci.* (2017) 11:224. 10.3389/fnbeh.2017.00224 29209180 PMC5701669

[52] Huster RJ Stevens S Gerlach AL Rist F. A spectralanalytic approach to emotional responses evoked through picture presentation. *Int J Psychophysiol.* (2009) 72:212–6. 10.1016/j.ijpsycho.2008.12.009 19135486

[53] Murphy FC Nimmo-Smith I Lawrence AD. Functional neuroanatomy of emotions: a meta-analysis. *Cogn Affect Behav Neurosci.* (2003) 3:207–33. 10.3758/cabn.3.3.207 14672157

[54] Winkler I Jager M Mihajlovic V Tsoneva T. Frontal eeg asymmetry based classification of emotional valence using common spatial patterns. *World Acad Sci Eng Technol.* (2010) 45:373–8. 10.5281/zenodo.1061729

[55] Cantero JL Atienza M Salas RM Gómez CM. Alpha EEG coherence in different brain states: an electrophysiological index of the arousal level in human subjects. *Neurosci Lett.* (1999) 271:167–70. 10.1016/S0304-3940(99)00565-0 10507695

[56] Härpfer K Spychalski D Kathmann N Riesel A. Diverging patterns of EEG alpha asymmetry in anxious apprehension and anxious arousal. *Biol Psychol.* (2021) 162:108111. 10.1016/j.biopsycho.2021.108111 33961931

[57] Armstrong T McClenahan L Kittle J Olatunji BO. Don’t look now! oculomotor avoidance as a conditioned disgust response. *Emotion.* (2014) 14:95–104. 10.1037/a0034558 24188060 PMC4040290

[58] Oosterwijk S. Choosing the negative: a behavioral demonstration of morbid curiosity. *PLoS One.* (2017) 12:e0178399. 10.1371/journal.pone.0178399 28683147 PMC5500011

[59] Fink-Lamotte J Platter P Stierle C Exner C. Mechanisms and effectiveness of imagery strategies in reducing disgust in contamination-related obsessive–compulsive disorder: comparing imagery rescripting, imagery self-compassion and mood-focused imagery. *Cogn Ther Res.* (2022) 46:747–63. 10.1007/s10608-021-10275-9

[60] Prause N Staley C Roberts V. Frontal alpha asymmetry and sexually motivated states. *Psychophysiology.* (2014) 51:226–35. 10.1111/psyp.12173 24460762

[61] Schöne B Schomberg J Gruber T Quirin M. Event-related frontal alpha asymmetries: electrophysiological correlates of approach motivation. *Exp Brain Res.* (2016) 234:559–67. 10.1007/s00221-015-4483-6 26537961

[62] Coan JA Allen JJB McKnight PE. A capability model of individual differences in frontal EEG asymmetry. *Biol Psychol.* (2006) 72:198–207. 10.1016/j.biopsycho.2005.10.003 16316717 PMC2835626

[63] Stewart JL Coan JA Towers DN Allen JJB. Resting and task-elicited prefrontal EEG alpha asymmetry in depression: support for the capability model. *Psychophysiology.* (2014) 51:446–55. 10.1111/psyp.12191 24611480 PMC3984363

[64] Prete G Capotosto P Zappasodi F Tommasi L. Contrasting hemispheric asymmetries for emotional processing from event-related potentials and behavioral responses. *Neuropsychology.* (2018) 32:317–28. 10.1037/neu0000443 29469582

[65] Halgren M Ulbert I Bastuji H Fabó D Erőss L Rey M The generation and propagation of the human alpha rhythm. *Proc Natl Acad Sci USA.* (2019) 116:23772–82. 31685634 10.1073/pnas.1913092116 PMC6876194

