# Supplementary material for: Ewww–Investigating the neural basis of disgust in response to naturalistic and pictorial nauseating stimuli
Source: Front Psychiatry. 2023 Jan 23;13:1054224. doi: 10.3389/fpsyt.2022.1054224 (PMC9899807; doi:10.3389/fpsyt.2022.1054224)
Supplement: Supplementary file 1 [file Data_Sheet_1.docx]

**Supplementary Material**

**Ewww - Investigating the neural basis of disgust in response to naturalistic and pictorial nauseating stimuli**

Gesa Berretz^a#^, Canan Dutschke^a^, Elodie Leonard^a^, Julian Packheiser^b^

^a^: Department of Biopsychology, Institute of Cognitive Neuroscience, Faculty of Psychology, Ruhr University Bochum, Bochum, Germany

^b^: Netherlands Institute for Neuroscience, Social Brain Lab, Amsterdam, The Netherlands

#: Corresponding author: Gesa Berretz

Telephone Number: +49 234 32 21453

Fax Number: +49 234 32 14377

E-Mail: Gesa.Berretz@rub.de

Address: Biopsychology, Ruhr University, Universitätsstraße 150, Room IB 6/109, 44780 Bochum, Germany.

Supplementary table S1. Descriptive data (means and SDs) of disgust ratings.

|  |  | Experienced disgust | Imagined discomfort |
| --- | --- | --- | --- |
| Pictorial | nauseating | 2.64 ± 2.12 | 3.09 ± 2.14 |
|  | neutral | 0.53 ± 0.62 | 0.50 ± 0.62 |
| Naturalistic | nauseating | 3.35 ± 2.12 | 3.09 ± 2.10 |
|  | neutral | 0.65 ± 0.58 | 0.58 ± 0.55 |

Supplementary table S2. ANOVA for perceived disgust.

|  | df | F | p | Partial η² |
| --- | --- | --- | --- | --- |
| Condition | 1 | 10.88 | 0.002 | 0.17 |
| Emotion | 1 | 108.93 | 0.000 | 0.66 |
| Condition * Emotion | 1 | 5.13 | 0.027 | 0.09 |

Supplementary table S3. ANOVA for imagined discomfort.

|  | df | F | p | Partial η² |
| --- | --- | --- | --- | --- |
| Condition | 1 | 0.10 | 0.760 | 0.00 |
| Emotion | 1 | 112.23 | 0.000 | 0.67 |
| Condition * Emotion | 1 | 0.11 | 0.743 | 0.00 |

Supplementary table S4. Descriptive data of AIs (means and SDs) for all participants.

|  |  | F3/4 | F5/6 | F7/8 | O1/2 |
| --- | --- | --- | --- | --- | --- |
| Pictorial | nauseating | 0.07 ± 0.19 | 0.04 ± 0.16 | 0.01 ± 0.22 | 0.00 ± 0.20 |
|  | neutral | 0.06 ± 0.14 | 0.06 ± 0.22 | 0.08 ± 0.18 | 0.01 ± 0.20 |
| Naturalistic | nauseating | 0.05 ± 0.22 | 0.04 ± 0.16 | 0.01 ± 0.24 | -0.05 ± 0.25 |
|  | neutral | 0.03 ± 0.22 | 0.03 ± 0.18 | 0.01 ± 0.22 | 0.01 ± 0.22 |

Supplementary table S5. Descriptive data of AIs (means and SDs) for disgusted participants.

|  |  | F3/4 | F5/6 | F7/8 | O1/2 |
| --- | --- | --- | --- | --- | --- |
| Pictorial | nauseating | 0.08 ± 0.22 | 0.04 ± 0.19 | -0.01 ± 0.23 | 0.02 ± 0.19 |
|  | neutral | 0.07 ± 0.16 | 0.06 ± 0.26 | 0.08 ± 0.20 | 0.03 ± 0.22 |
| Naturalistic | nauseating | 0.05 ± 0.23 | 0.02 ± 0.17 | -0.02 ± 0.25 | -0.07 ± 0.26 |
|  | neutral | 0.04 ± 0.25 | 0.02 ± 0.20 | 0.00 ± 0.21 | 0.03 ± 0.22 |


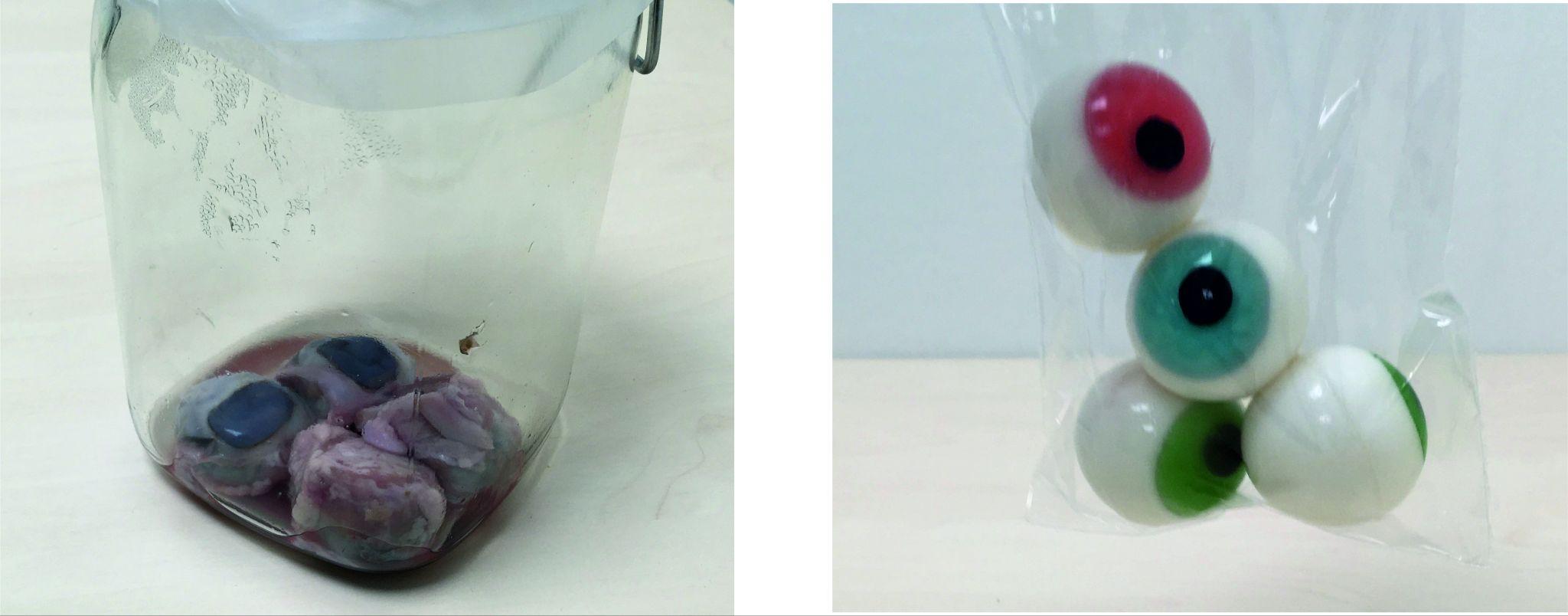


Supplementary Figure 1. Pictures of sheep eyes (left) and corresponding candy eyeballs (right) used in the study. Sheep eyes were acquired at a local butcher and kept and presented in a sealed glass jar. Candy eyes were kept in a sealed plastic bag.


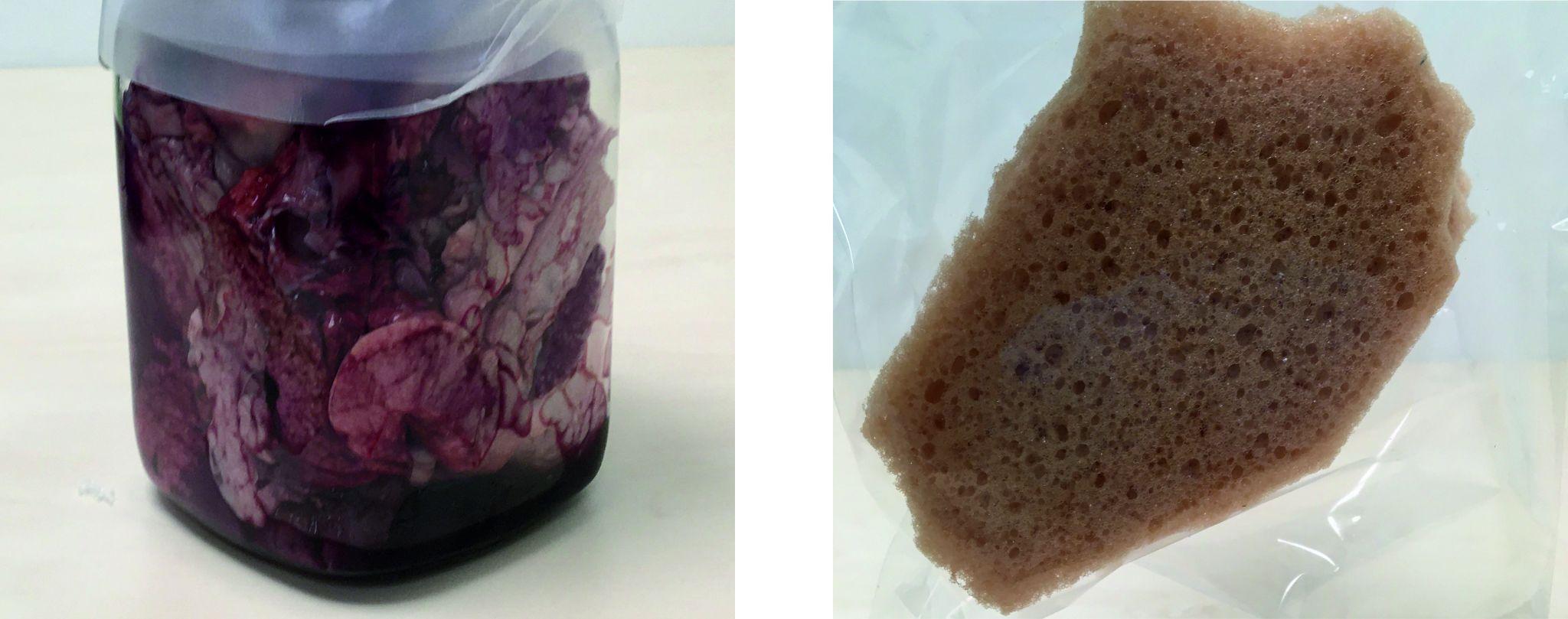


Supplementary Figure 2. Pictures of sheep lung (left) and corresponding sponge (right). Sheep lungs were acquired at a local butcher and kept and presented in a sealed glass jar. The sponge was kept in a sealed plastic bag.


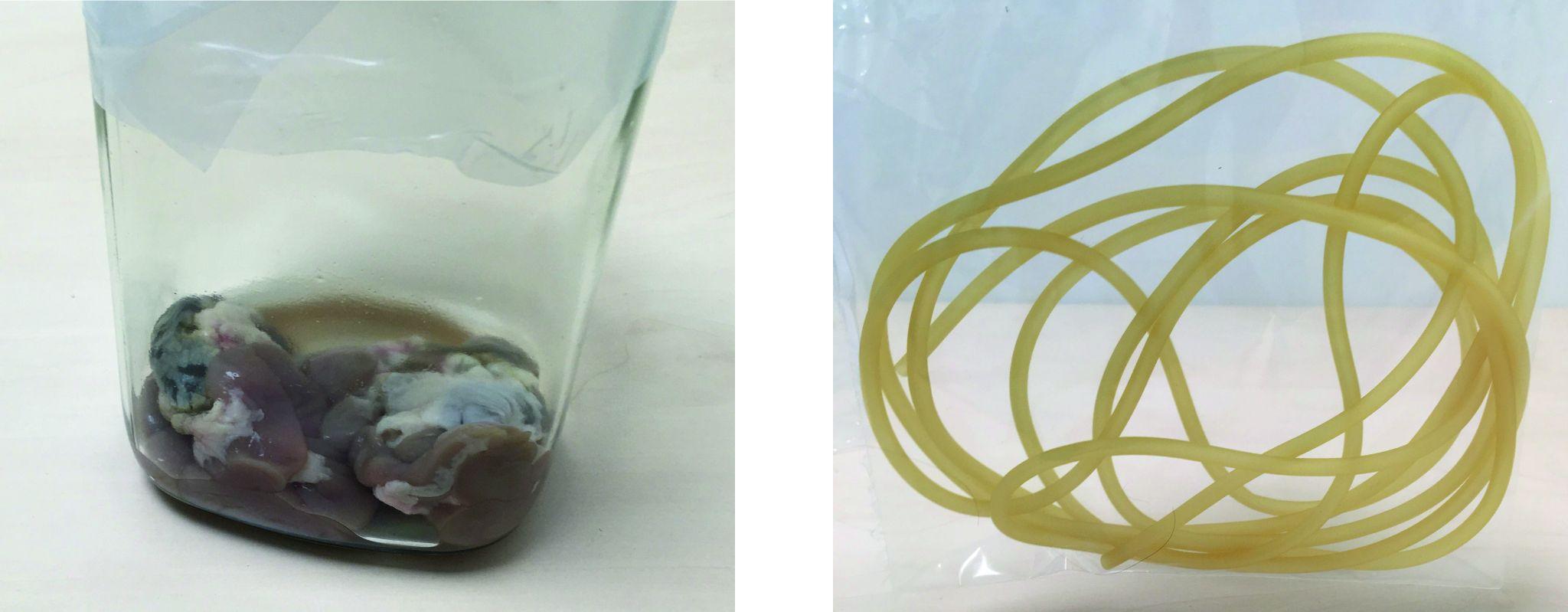


Supplementary Figure 3. Pictures of sheep intestine(left) and corresponding rubber tubing (right) used in the study. Sheep intestines were acquired at a local butcher and kept and presented in a sealed glass jar. Rubber tubes were kept in a sealed plastic bag.


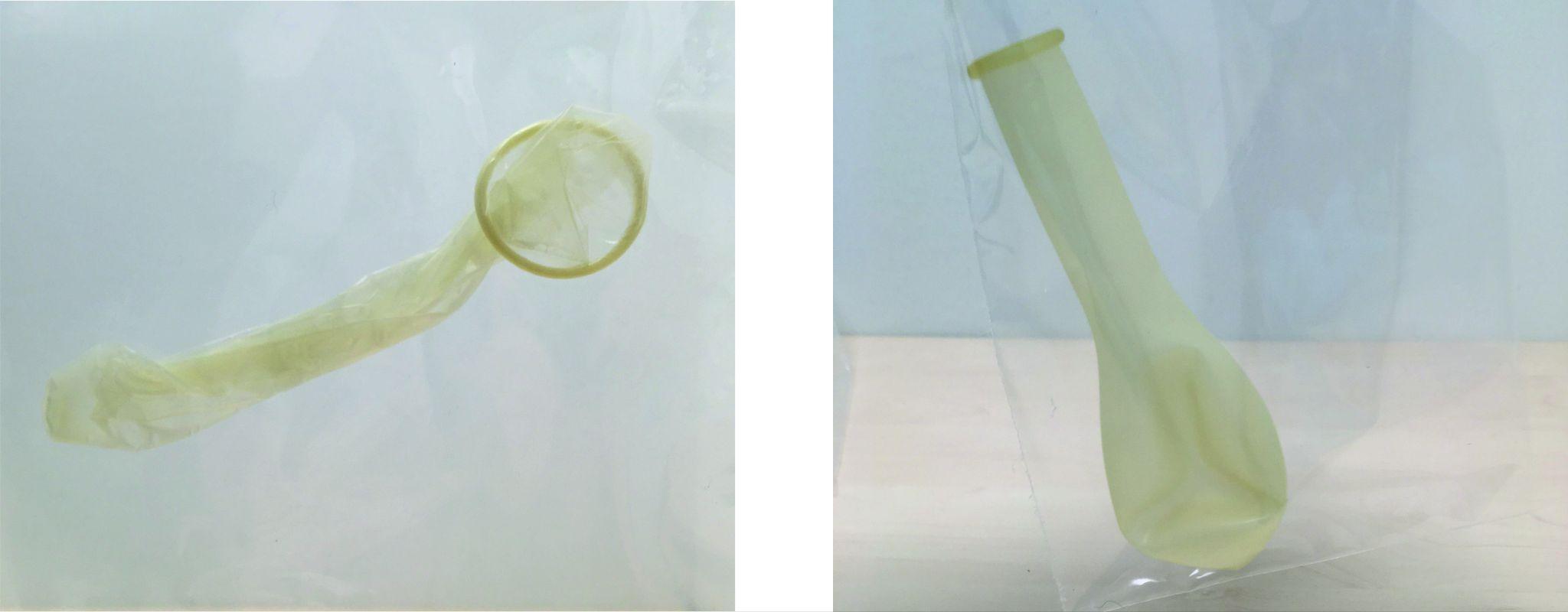


Supplementary Figure 4. Pictures of a used condom (left) and corresponding balloon (right). The condom was a standard condom removed from its packaging. Semen was simulated with yogurt. Both stimuli were kept in sealed plastic bags.


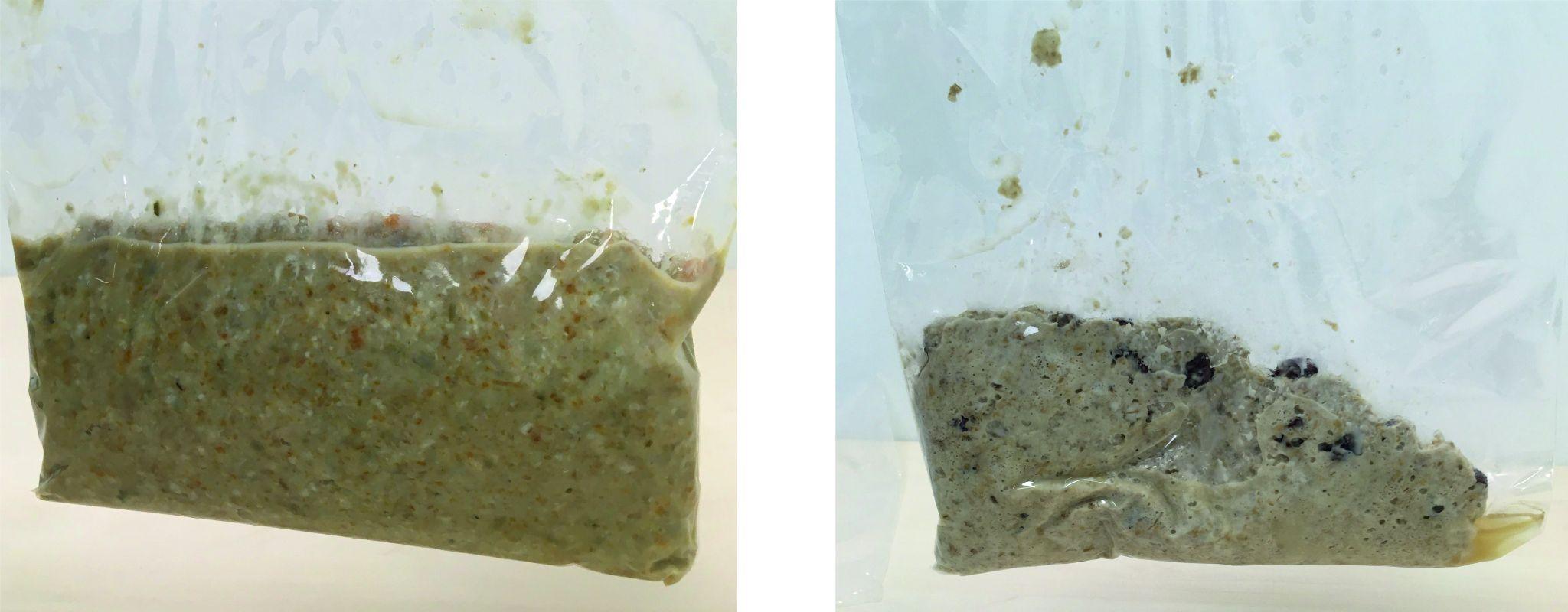


Supplementary Figure 5. Pictures of vomit (left) and corresponding oatmeal (right). As obtaining real vomit was not possible, fake vomit was created by mixing oats, canned pea soup, carrots, and water. Oatmeal was prepared with oats, milk, and raisins. Both stimuli were kept in sealed plastic bags.


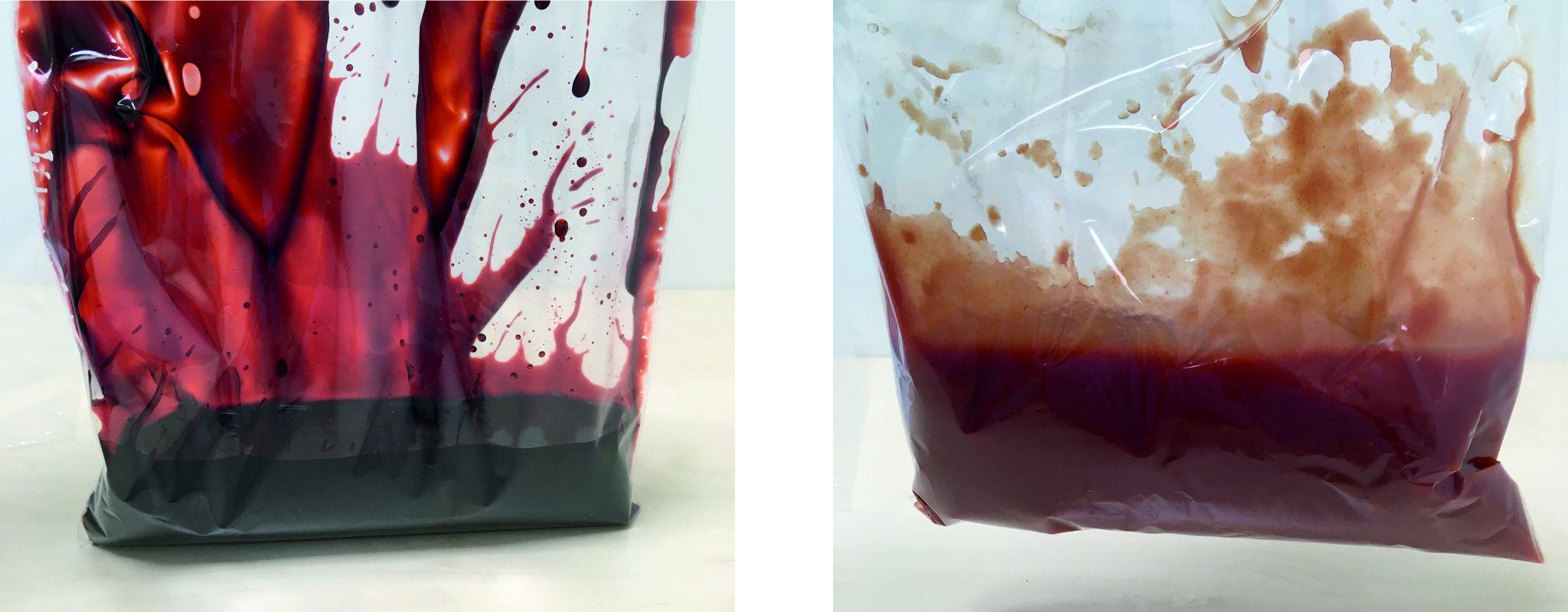


Supplementary Figure 6. Pictures of blood (left) and corresponding tomato sauce (right). As obtaining a large amount of real blood was not possible, we utilized fake blood used in film. Tomato sauce was prepared from pureed tomatoes and water. Both stimuli were kept in sealed plastic bags.


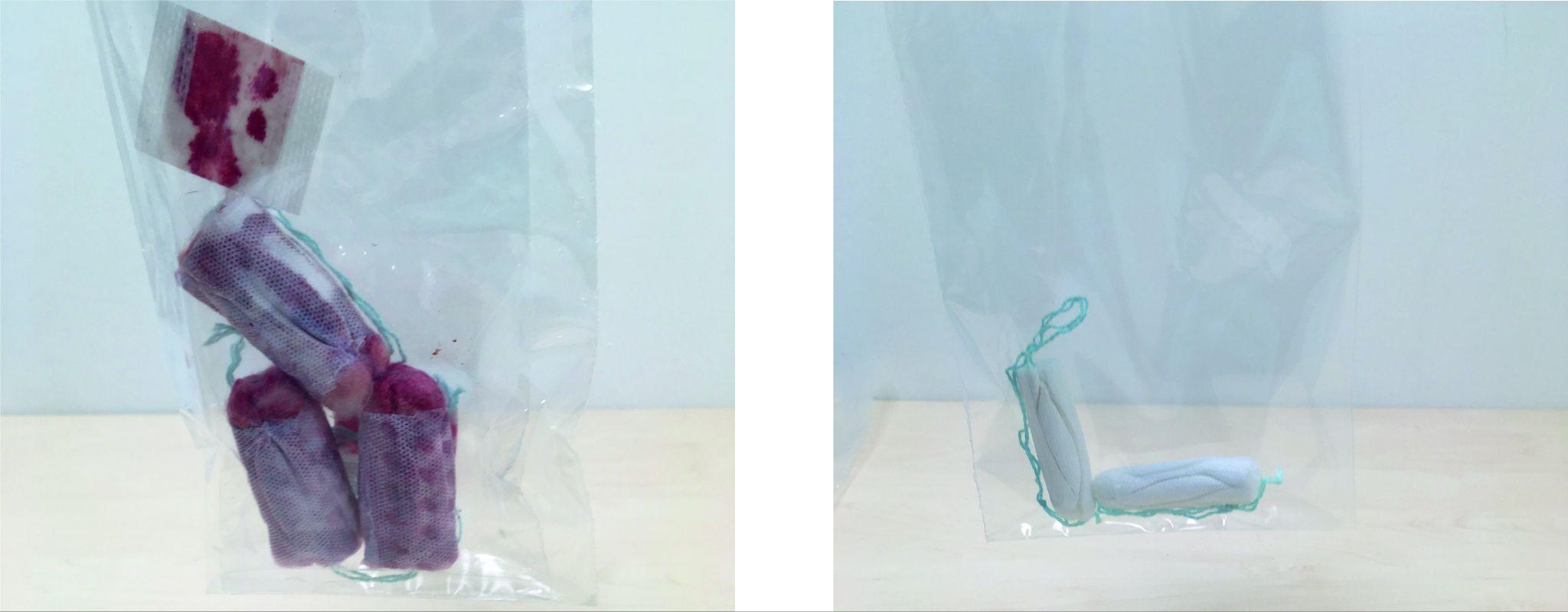


Supplementary Figure 7. Pictures of used (left) and corresponding unused hygiene products (right). used hygiene products were drenched in real blood drawn from an underarm vein of GB. Unused tampons were unwrapped from the plastic packaging. Both stimuli were kept in sealed plastic bags.


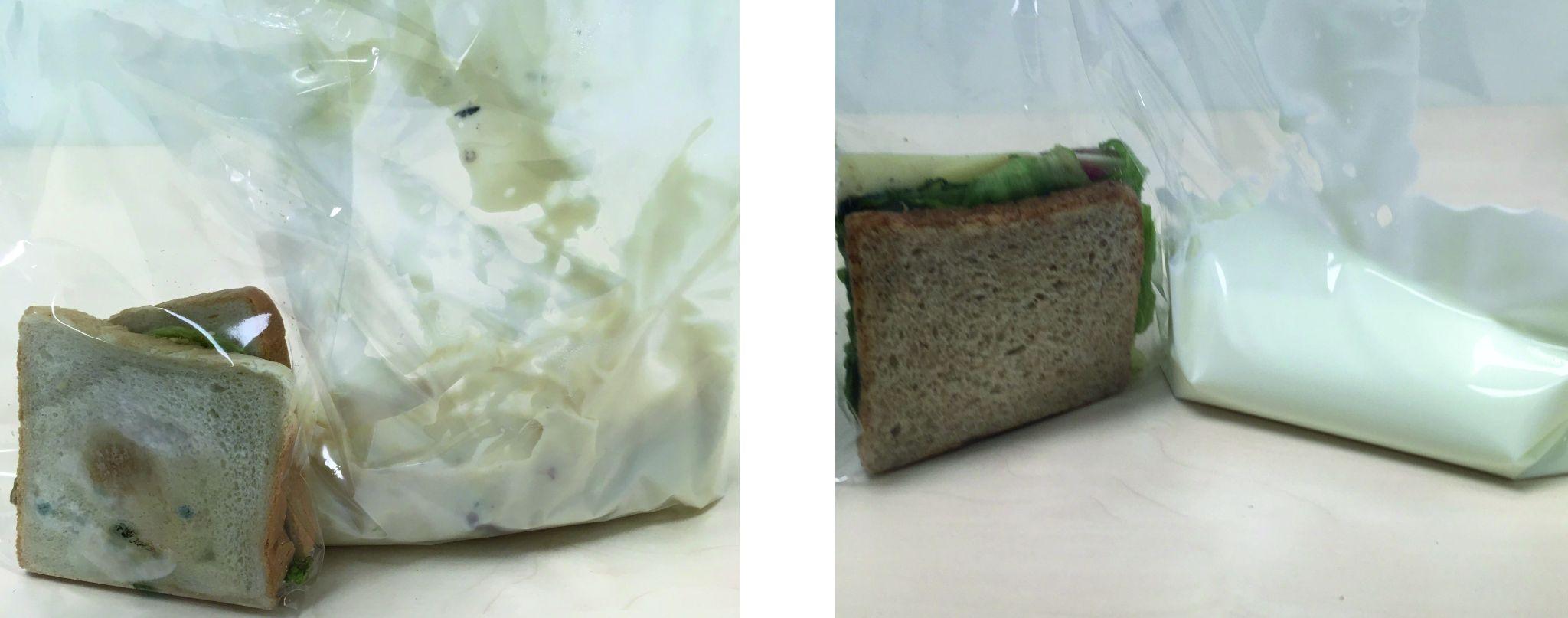


Supplementary Figure 8. Pictures of spoiled (left) and fresh food (right). Fresh food consisted of a sandwich with cheese, tomatoes, and salad as well as plain yogurt. Spoiled food consisted of the same ingredients having been kept at room temperature for two weeks to cause mold. Both stimuli were kept in sealed plastic bags.


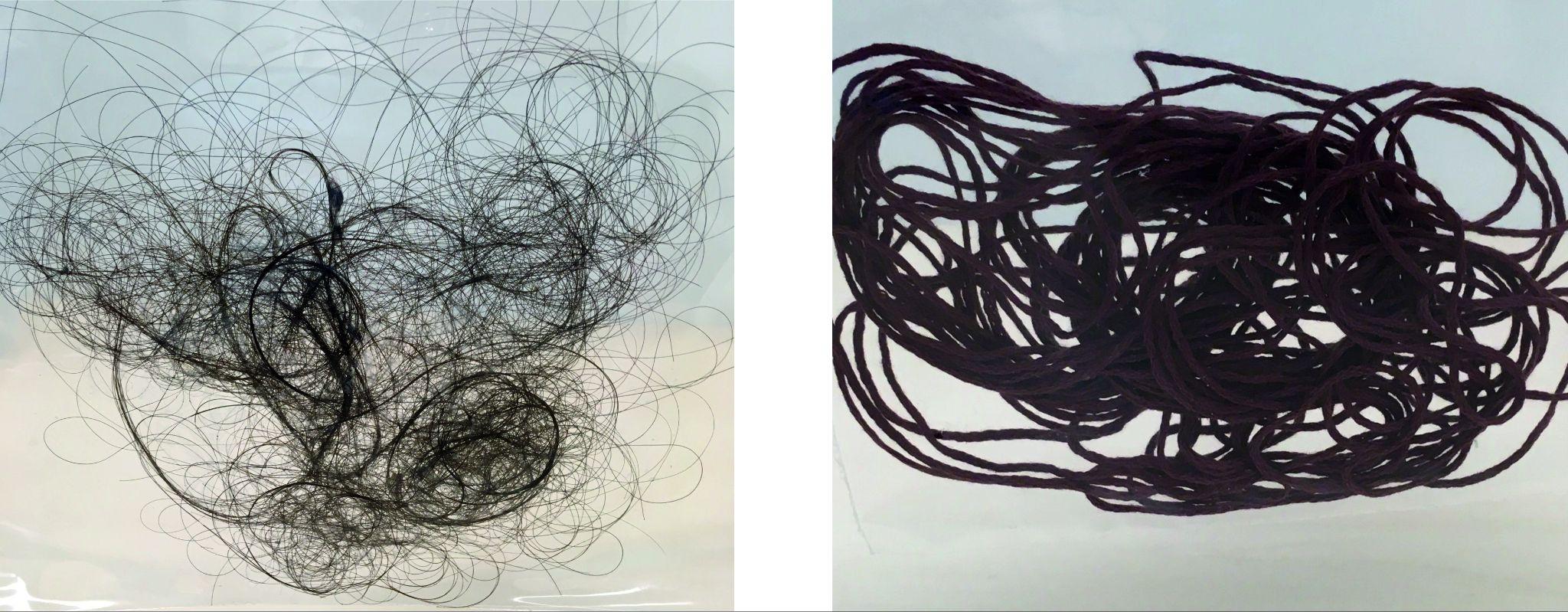


Supplementary Figure 9. Pictures of hair from the shower drain (left) and corresponding brown yarn (right). A student assistant from the Ruhr University Bochum provided hair. Both stimuli were kept in sealed plastic bags.


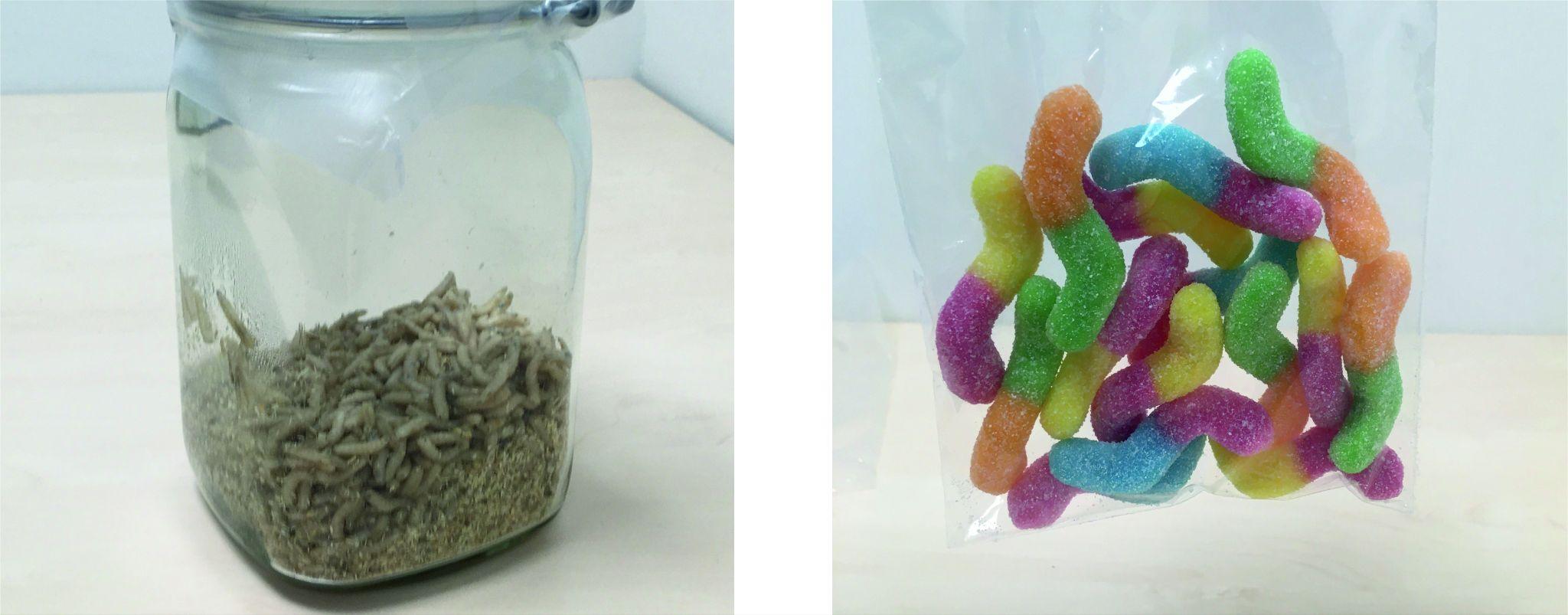


Supplementary Figure 10. Pictures of dead mealworms (left) and corresponding candy worms (right). Dead mealworms were acquired t a local fish food shop. Gummy worms were removed from the packaging. Both stimuli were kept in sealed plastic bags.
